# Combined RNA-seq and RAT-seq mapping of long noncoding RNAs in pluripotent reprogramming

**DOI:** 10.1038/sdata.2018.255

**Published:** 2018-11-20

**Authors:** Zhonghua Du, Lin Jia, Yichen Wang, Cong Wang, Xue Wen, Jingcheng Chen, Yanbo Zhu, Dehai Yu, Lei Zhou, Naifei Chen, Shilin Zhang, Ilkay Celik, Ferhat Ay, Sujun Gao, Songling Zhang, Wei Li, Andrew R. Hoffman, Jiuwei Cui, Ji-Fan Hu

**Affiliations:** 1 Stem Cell and Cancer Center, First Hospital, Jilin University, Changchun, Jilin 130061, P.R. China; 2 Stanford University Medical School, VA Palo Alto Health Care System, Palo Alto, CA 94304, USA; 3 La Jolla Institute for Allergy and Immunology, La Jolla, California 92037, USA

**Keywords:** Long non-coding RNAs, Regenerative medicine

## Abstract

Pluripotent stem cells hold great investigative potential for developmental biology and regenerative medicine. Recent studies suggest that long noncoding RNAs (lncRNAs) may function as key regulators of the maintenance and the lineage differentiation of stem cells. However, the underlying mechanisms by which lncRNAs affect the reprogramming process of somatic cells into pluripotent cells remain largely unknown. Using fibroblasts and induced pluripotent stem cells (iPSCs) at different stages of reprogramming, we performed RNA transcriptome sequencing (RNA-Seq) to identify lncRNAs that are differentially-expressed in association with pluripotency. An RNA reverse transcription-associated trap sequencing (RAT-seq) approach was then utilized to generate a database to map the regulatory element network for lncRNA candidates. Integration of these datasets can facilitate the identification of functional lncRNAs that are associated with reprogramming. Identification of lncRNAs that regulate pluripotency may lead to new strategies for enhancing iPSC induction in regenerative medicine.

## Background & Summary

Pluripotent stem cells, whether directly isolated from blastocysts or induced by Oct4-Sox2-Klf4-c-Myc (OSKM) reprogramming, have significant therapeutic potential for regenerative medicine^1–3^. Somatic cells, such as fibroblasts, can be reprogrammed *in vitro* into induced pluripotent stem cells (iPSCs) through the delivery of pluripotency-related transcription factors (OSKM)^4,5^. This reprogramming process converts somatic cells into an embryonic-like state, offering the opportunity to study cellular differentiation and ultimately, to develop therapies for regenerative medicine^6–8^. However, successful reprogramming of somatic cells into iPSCs is extremely inefficient and time-consuming. The discovery of potential epigenetic barriers to reprogramming may reveal new approaches for enhancing iPSC induction.

Using exogenous OSKM factors to initiate reprogramming is a multistep process^9^, in which somatic cells must overcome a series of epigenetic roadblocks to acquire pluripotency^10,11^. It is now clear that reprogramming to pluripotency is associated with a fundamental epigenetic reset of the chromatin landscape^12,13^, which is deemed critical for activation of the transcriptional network that is associated with pluripotency, while silencing other genes that are specifically transcribed in differentiated tissues.

Noncoding RNAs are critical players in organizing the regulatory networks that establish or remove roadblocks in this reprogramming process^14–17^. Long non-coding RNAs (lncRNAs), which are >200 nucleotides in length and lack apparent open reading frames, may control gene activity at a transcriptional level via either *cis* or *trans* mechanisms^18,19^. Thousands of lncRNAs have been identified through comparative transcriptomics, particularly RNA sequencing (RNA-seq), in embryonic stem cells (ESCs)^20,21^. However, only a few of these lncRNAs have been functionally characterized in the regulation of pluripotency or reprogramming.

In this communication, we present two high-throughout sequencing datasets that may be useful in functionally mapping those lncRNAs that are associated with pluripotency. We collected fibroblasts and iPSCs that were in the process of reprogramming. RNA transcriptome sequencing (RNA-seq) was initially performed for these two types of cells to help identify lncRNAs that are differentially expressed in reprogrammed cells. It was assumed that some of these lncRNAs might play critical roles in the establishment of an ES cell-specific transcriptional network. An RNA reverse transcription-associated trap sequencing (RAT-seq) approach was then utilized to map the regulatory element network for these lncRNA candidates. The combination of RNA-seq and RAT-seq datasets will allow investigators to identify pluripotency-associated lncRNAs.

## Methods

### Reprogramming of mouse fibroblasts towards pluripotency

**Figure 1:**
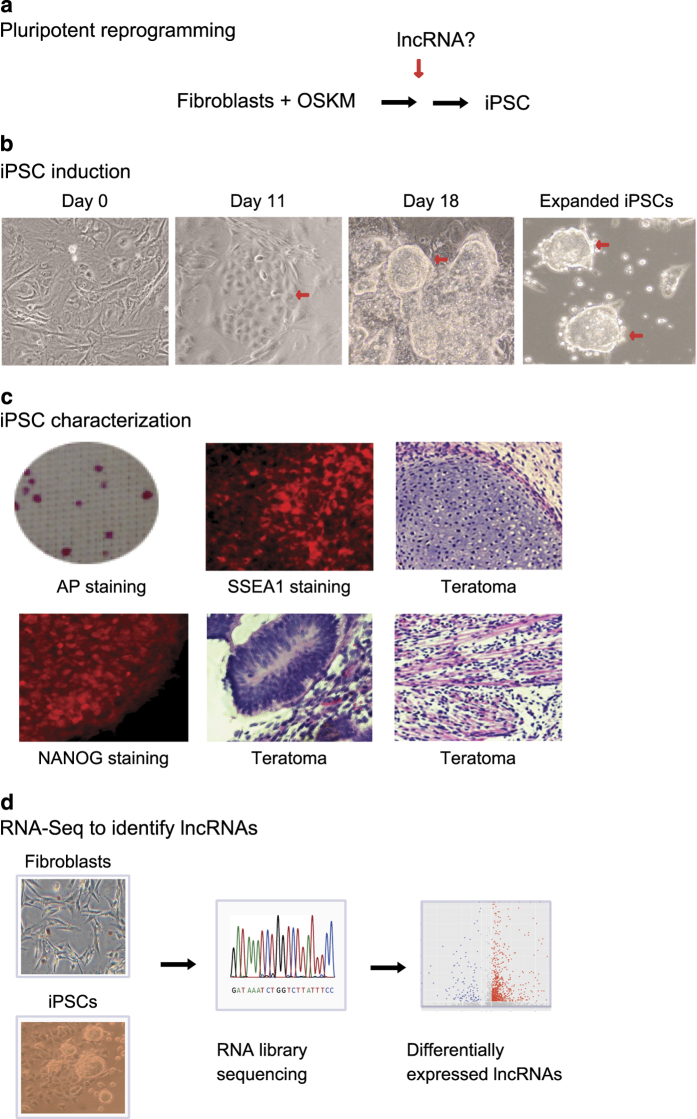
Reprogramming of fibroblasts into pluripotent stem cells. (**a**) Schematic diagram of pluripotent reprogramming. Fibroblasts were transfected with lentiviruses carrying Oct4-Sox2-Klf3-c-Myc (OSKM) transcription factors and were induced into pluripotency. iPSC: induced pluripotent stem cell; lncRNA: long noncoding RNA. (**b**) Typical images of iPSC reprogramming. After OSKM transfection, cells were cultured on MEF feeder cells in mouse stem cell culture. Cell images were taken at different stages of reprogramming. Red arrows: dynamic changes of cell morphology. (**c**) Characterization of iPSCs. After induction, iPSC colonies were stained for alkaline phosphatase, a stem cell marker. At the end of reprogramming, iPSC colonies were expanded on MEF feeder cells. After expansion, stable iPSCs were examined for pluripotency by immunochemical staining of pluripotent biomarkers, including NANOG and SSEA4. iPSCs were used for further testing of teratoma formation in nude mice. (**d**) Schematic diagram of RNA-seq for fibroblasts and iPSCs. Thousands of RNAs were found to be differentially expressed after reprogramming.

It was assumed that some lncRNAs exert a critical role in the process of fibroblast reprogramming (Fig. 1a). To identify lncRNAs that are associated with pluripotency, mouse fibroblasts were first reprogrammed with Oct4-Sox2-Klf4-c-Myc (OSKM) lentiviruses as previously described^22,23^. Lentiviruses were packaged in 293 T cells using lipofectamine 2000 (Invitrogen, CA). The virus-containing supernatants were collected and concentrated with Centrifugal Filter Units (Amicon Ultra-15, Millipore, MA). Fibroblasts were seeded in 6-well plates and were infected with lentiviruses using 8 μg/ml polybrene. After viral infection, the cells were transferred to 100 mm dishes on MEF feeder cells and were cultured in ES medium (DMEM/F12 supplemented with 20% KSR, 10 ng/ml Leukemia inhibitory factor (LIF, Sigma, MO), 10 ng/ml β-FGF (PeproTech, NJ), 0.1 mM β-mercaptoethanol, L-Glutamine, and 1 × 10^−4^ M non-essential amino acids^24^. The visible iPSC colonies were selected as previously described (Fig. 1b)^22,23^. After continuous expansion, iPSCs were further characterized for pluripotency. Fibroblasts were collected as the control cells. Cells that expressed the virally-produced OSKM factors but had not been converted into iPSCs were collected as “un-reprogrammed control cells.” As previously described^23^, in these unreprogrammed cells, the virally-produced OSKM factors bind to their target genes, but they fail to activate these endogenous target genes to initiate reprogramming. We have shown that this failure to reprogram cells into iPSCs was related to the lack of intrachromosomal loops that bring the distal enhancers close to the promoters to activate them to initiate reprogramming. The formation of intrachromosomal loops in pluripotency-associated genes constitutes a critical epigenetic barrier that must be overcome for cell reprogramming to occur^25^.

### Characterization of isolated iPSCs

iPSC colonies were expanded and stained for the alkaline phosphatase (AP) stem cell marker using the Alkaline Phosphatase Detection Kit (SCR004, Millipore, CA) following the manufacturer’s instructions^22,23^. Briefly, the cells were fixed in 4% paraformaldehyde/PBS for 1–2 min, rinsed with PBS and then incubated with staining solution in the dark at room temperature. Colonies of iPSC cells expressing AP were assessed using a microscope-mounted camera (Fig. 1c).

The isolated iPSCs were also examined for the expression of the pluripotency markers NANOG and SSEA1 using Fluorescent Mouse ES/iPS Cell Characterization Kit (SCR077, Millipore, CA), as previously described^22,23^. Cells were fixed using 4% paraformaldehyde/PBS for 10–15 min and rinsed with PBS, then permeabilized and blocked with 0.1% Triton X-100/PBS containing 3% BSA for 30 min. After washing with PBS, cells were incubated with Cy3 labeled antibodies overnight at 4 °C (SSEA1, MAB4301C3; NANOG, MABD24C3). After washing three times with PBS, samples were counterstained with Hoechst 33258 (Invitrogen). Negative controls were stained without the use of primary antibodies. Fluorescence images were acquired with a Zeiss AxioCam Camera.

The pluripotency of iPSC clones was further confirmed by a teratoma assay^22,24^. iPSC cells (5 × 10^6^ cells/ml) were resuspended in 200 μl matrigel (BD Biosciences, CA) and then injected subcutaneously into the dorsal flank of SCID mice. Teratomas were fixed in 4% paraformaldehyde, dissected and embedded in paraffin. The sections were stained with hematoxylin and eosin (HE) for histological analysis. Taken together, these assays confirmed the pluripotency of the isolated iPSCs (Fig. 1c).

### RNA library sequencing

After confirmation of pluripotency, Illumina RNA library sequencing was used to identify RNAs and lncRNAs that are differentially expressed in the reprogrammed cells (Fig. 1d). Total RNA was extracted using TRIZOL Reagent (15596-018, Invitrogen, CA) following the manufacturer’s instructions. The isolated RNAs were checked for RNA integrity by an Agilent Bioanalyzer 2100 (Agilent technologies, CA, US). Total RNA was further purified by RNAClean XP Kit (A63987, Beckman Coulter, CA). RNase-Free DNase I (79254, QIAGEN, CA) was used to remove any contaminating DNA.

**Figure 2:**
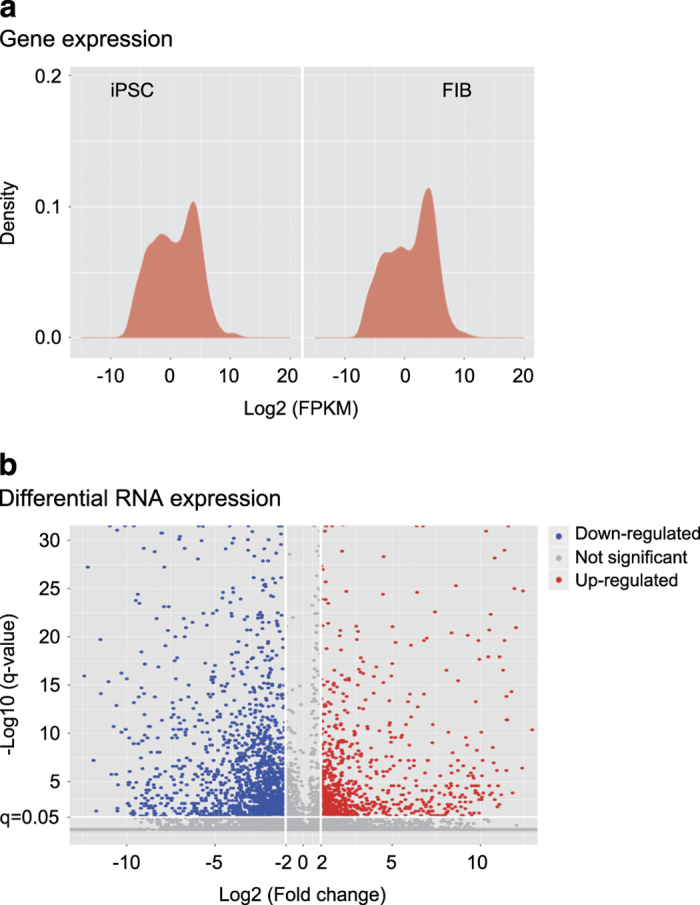
Differential expression of RNAs by RNA-seq. (**a**) RNA-seq expression patterns between iPSCs and fibroblasts. FIB (FBC): fibroblasts; iPSC: induced pluripotent stem cells. (**b**) Differential expression of RNAs between iPSCs and fibroblasts (FIB or FBC). Blue dots: down-regulated RNAs; red dots: upregulated RNAs.

Ribosomal RNA was removed by TruSeq Stranded Total RNA LT -(Ribo-Zero TM Gold)-Set A/B (#RS-122-2301/2302, Illumina, CA). RNAs were then fragmented into small pieces using a fragmentation reagent. The fragmented RNAs were subjected to first-strand cDNA synthesis using random hexamer-primed reverse transcription (18064014, SupperScript II reverse Transcriptase, Invitrogen, CA), followed by second-strand cDNA synthesis (Q32850, Qubit dsDNA HS Assay Kit, Invitrogen, CA). The cDNA fragments were 3′ adenylated and ligated with adaptors for PCR amplification for library construction. The library quality was checked using Agilent2100, producing on average 370–380 bp fragments, including adapters. The libraries were clustered on Illumina cBot Instrument and pair-sequenced (Data Citation 1). Fig. 2 shows the density and volcano plots for RNA-seq datasets from iPSCs and fibroblasts.

### RAT-seq to identify lncRNA target gene network

**Figure 3:**
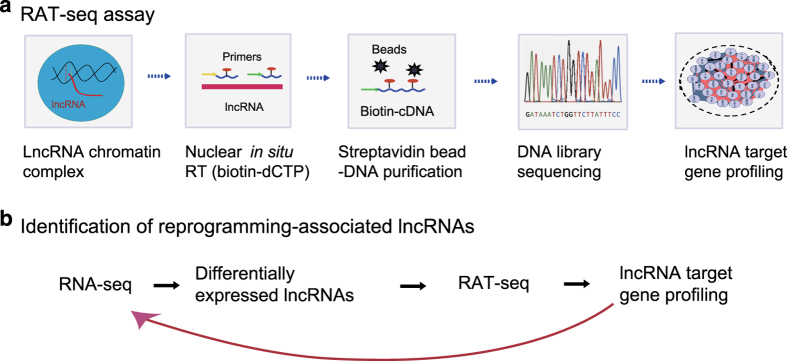
RAT-seq to identify the target genes of lncRNA candidates. (**a**) Schematic diagram of the RNA *in situ* reverse transcription-associated trap (RAT) assay. After fixation with formaldehyde, a given lncRNA was *in situ* reverse transcribed using lncRNA-specific primers and biotin-dCTP. The lncRNA-biotin cDNA chromatin complexes are pulled down with streptavidin beads and the chromatin complex DNAs to which the lncRNA binds are isolated for library sequencing. RAT-seq will map the gene targets genome-wide for the lncRNA candidate. (**b**) Integration of RNA-seq and RAT-seq to study the reprogramming-associated lncRNAs. Firstly, RNA-seq is used to identify lncRNAs that are differentially transcribed in the process of reprogramming. Then, a RAT-seq assay is performed to identify potential lncRNAs of interest. The RAT-seq data will help determine if an lncRNA is able to interact with multiple target genes, particularly key stem cell core factor genes. The detection of physical interactions, particularly in the gene promoter and enhancer areas, might suggest that the lncRNA is a component of the chromatin complex, and may be involved in the regulation of stem cell fate.

Bioinformatic analysis of RNA-Seq data often generates a list of thousands of differentially expressed lncRNAs. The major challenge is to identify the key functional lncRNAs from these large RNA-Seq pools. We modified an “RNA reverse transcription-associated trap sequencing” (RAT-seq) method that we had previously established in our lab^26,27^, and attempted to map the gene targets genome-wide to discover potential lncRNA candidates (Fig. 3a).

We hypothesized that a pluripotency-associated lncRNA candidate should have the potential to regulate multiple stem cell core factor genes and/or the pathway genes that are critical for reprogramming. Thus, we used RAT-seq data to narrow the number of lncRNA candidates to those lncRNAs that play a critical role in the regulation of the pluripotency-specific transcriptional network. By integrating the RNA-seq and RAT-seq datasets, we attempted to identify potential lncRNA candidates that are not only differentially expressed after reprogramming, but also have the capacity to bind to the regulatory elements of multiple pluripotency-associated pathway genes and stem cell core factor genes (Fig. 3b), such as Oct4, Sox2, and Nanog.

We performed RAT-seq for lncRNA NONMMUT043505 (pluripotency-associated transcript 10, *Platr10*;^28^ Oct4-Sox2 coating long noncoding RNA 8, *Osclr8*). In the RAT-seq assay^26,27^, cells were cross-linked with 2% formaldehyde and lysed with cell lysis buffer (10 mM Tris [pH 8.0], 10 mM NaCl, 0.2% NP-40, 1 × protease inhibitors). Nuclei were suspended in 1 × reverse transcription buffer in the presence of 0.3% sodium dodecyl sulfate (SDS) and incubated at 37 °C for 1 h. Triton X-100 was then added to a final concentration of 1.8% to sequester the SDS. Gene strand-specific reverse transcription was performed at 65 °C using Maxima Reverse Transcriptase (Thermo Fisher Scientific, CA) with lncRNA-specific antisense oligonucleotides and biotin-dCTP. In this example, three RAT oligonucleotides were used to prepare the RT reaction for lncRNA NONMMUT043505, including JH4023: 5′-TGGGACAGTCTCTGGATGGCCT-3′; JH4397: 5′-CATGATGCTGGAGAGGTAGCT-3′; AND JH4398: 5′-AGAGGGAATCTAGGCAGGTAG-3′. For the RAT control, reverse transcription was performed in parallel using random oligonucleotides JH5849: 5′-ATGGACTGATGATCTTATGC-3′ and JH5850 : 5′-TACATAGTAGATCAGATACT-3′. After 50 min of reverse transcription, the reaction was stopped by adding 4 μl 0.5M EDTA.

After nuclear lysis, the chromatin complex was subjected to sonication for 180 s (10 s on and 10 s off) on ice with a Branson sonicator with a 2-mm microtip at 40% output control and 90% duty cycle settings. The biotin-lncRNA-cDNA/chromatin DNA complexes were pulled down with streptavidin magnetic beads (Invitrogen, CA). After reversing the cross-links and washing with 10 mg/ml proteinase K at 65 °C overnight, the chromatin complexes were treated with 0.4 μg/ml RNase A for 30 min at 37 °C. The genomic DNA that interacted with the lncRNA was extracted and digested by Dpn I and ligated with the NEBNext adaptors (NEBNext® ChIP-Seq Library Prep Master Mix Set for Illumina) to construct the library. The library DNAs were subjected to Illumina sequencing by Shanghai Biotechnology (Shanghai, PRC).

### Code availability

The following software versions were used for quality control and data analysis:

FastQC (v0.11.2): bioinformatics.babraham.ac.uk/projects/fastqc/

Seqtk: https://github.com/lh3/seqtk

TopHat (version:2.0.9)^29^

Cufflinks (version:2.2.1)^30^

Cuffdiff:^30^

Fastx (version:0.0.13): http://hannonlab.cshl.edu/fastx_toolkit/index.html

Bowtie (version:0.12.8)^31^

MACS2 (version:2.1.1)^32^

MEME suite^33^

DiffBind^34^

VENN program: http://bioinformatics.psb.ugent.be/webtools/Venn/.

## Data Records

The RNA-Seq data for iPSCs and fibroblasts were deposited in NIH GEO databases (Data Citation 1). The RAT-seq data generated in this study, including lncRNA NONMMUT043505, the RAT random control, and the IgG control were deposited in NIH GEO databases in NIH GEO databases (Data Citation 2). The FastQ format data will serve as the raw sequencing data for further downstream processing. The processed data (bedgraph) were also deposited at NCBI Gene Expression Omnibus (Data Citations 1, 2).

## Technical Validation

### RNA-seq data for cells that were collected in reprogramming

Table 1 lists the quality of two RNA samples prepared from iPSCs and fibroblasts collected during the process of reprogramming.

After removal of ribosomal RNA, RNAs were used for library construction. The library quality was checked using Agilent2100, producing on average 370-380 bp fragments including adapters. Table 2 lists the quality of the libraries. The libraries were clustered on an Illumina cBot Instrument and pair-sequenced.

The RNA libraries were sequenced on Illumina Hiseq2100, generating 145,153,730 raw reads for iPSCs and 148,885,396 raw reads for fibroblasts. After filtering low quality reads, a total of 119,914,499 clean reads were obtained for iPSCs and 123,939,088 clean reads for fibroblasts. After Seqtk filtering, a total of 120 million clean reads and 124 million clean reads were mapped to the mouse genome (genome version: mm10, GRCm38.p4 (ftp://ftp.ensembl.org/pub/release-83/fasta/mus_musculus/dna/Mus_musculus.GRCm38.dna.primary_assembly.fa.gz) for mRNAs and lncRNAs using the STAR software^35^. Table 3 lists the mapping rate for each sample.

Gene counts were normalized to the values of Reads Per Kilobase of transcript per Million mapped reads (RPKM). Cuffdiff was used to calculate the differentially expressed RNAs when the fold-change was >2 and p <0.05 with an unpaired two-sided t-test (Fig. 2).

### RAT-seq to map the lncRNA target gene network

We proposed to use the RAT-seq to validate whether the RNA-seq identified RNAs that are critical for reprogramming (Fig. 3b). As an example, we performed RAT-seq for lncRNA NONMMUT043505. After sequencing, the adapter sequences were removed from the raw data using Illumina annotated adapter sequences with parameter ILLUMINACLIP:2:30:10 and the low quality data were filtered with parameters SLIDINGWINDOW:4:20 MINLEN:36, using Trimmomatic software^36^. More than 44 million Single-end reads of 50 bp length from the RAT-seq protocol were then mapped to the mouse genome (genome version: mm10) using the STAR software^35^ with default parameters. By combining RNA-Seq and RAT-seq datasets, it is possible to identify lncRNA candidates that may be functionally associated with pluripotency.

## Usage Notes

The RNA-seq dataset presented in this communication identifies thousands of lncRNA that are differentially expressed after reprogramming (Data Citation 1). It is important to determine which, if any, of these lncRNA play a role in the maintenance of pluripotency in stem cells. Our strategy was to perform the RAT-seq assay to focus on those lncRNAs that are not only differentially expressed after reprogramming, but are also able to bind to regulatory elements, such as promoters and enhancers, of core stem cell factors or pathway genes related to pluripotency (Data Citation 2). These RAT-seq data can also be used to examine whether a particular lncRNA function through epigenetic mechanisms, including alterations in chromatin three-dimensional structure, DNA methylation, histone modifications, and enhancer RNAs.

## Additional Information

**How to cite this article**: Zhonghua, D. *et al*. Combined RNA-seq and RAT-seq mapping of long noncoding RNAs in pluripotent reprogramming 2011–2013. *Sci. Data*. 5:180255 doi: 10.1038/sdata.2018.255 (2018).

**Publisher’s note**: Springer Nature remains neutral with regard to jurisdictional claims in published maps and institutional affiliations.

## Supplementary Material



## Figures and Tables

**Table 1:** Quality of the isolated RNAs.

No.	Sample Name	Con. (μg/μL)	Vol. (μL)	Amount (μg)	A260/ 280	2100 Result	Result	
						RIN^∗^		28S/18S
1	FIB	178.9	100	17.89	1.93	9	11.6	A3
2	PSC	700	150	105	2.01	9.3	1.8	A3
^∗^RIN: RNA integrity number; FIB (FBC): fibroblasts; PSC: iPSCs								

**Table 2:** The quality of the libraries.

Sample Name	Seq type	Orientation	Raw read (M)	Raw bases(G)	Q20 ratio (%)
FIB	RNA	Forward/Reverse	148.9	22.3	96.22
PSC	RNA	Forward/Reverse	145.1	21.7	96.64
FIB (FBC): fibroblasts; PSC: iPSCs					

**Table 3:** Mapping of the RNA-Seq data.

ID	All reads	Mapped reads	Mapped Pair Reads	Mapped Broken-pair reads	Mapped Unique reads	Mapped Multi reads	Mapping Ratio
FIB	117,507,104	104,053,152	96,013,068	8,040,084	101,884,006	2,169,146	88.55%
PSC	114,282,430	98,053,582	90,115,020	7,938,562	95,214,266	2,839,316	85.80%
FIB (FBC): fibroblasts; PSC: iPSCs							
